# Identifying optimal locations for automated external defibrillators (AED) in Freiburg: development and validation of a machine learning model based on demographic and infrastructural data

**DOI:** 10.1186/s12873-025-01441-3

**Published:** 2025-12-13

**Authors:** Julian Ganter, Hannah Bakker, Stefan Nickel, Elisa-Sophie Reichling, Alicia Wittmer, Niklas Werner, Thomas Brucklacher, Robert Wunderlich, Georg Trummer, Hans-Jörg Busch, Michael Patrick Müller

**Affiliations:** 1 Department of Anaesthesiology and Critical Care, Medical Center - University of Freiburg, Faculty of Medicine, University of Freiburg, Freiburg, Germany; 2 Health Care Lab, Karlsruhe Service Research Institute (KSRI), Karlsruhe Institute of Technology (KIT), Karlsruhe, Germany; 3 Region of Lifesavers [Region der Lebensretter e.V.], Freiburg, Germany; 4 University Department of Anesthesiology and Intensive Care Medicine, University Hospital Tübingen, Eberhard Karls University, Tübingen, Germany; 5 Department of Emergency Medicine, Medical Center - University of Freiburg, Faculty of Medicine, University of Freiburg, Freiburg, Germany; 6 Department of Anaesthesiology, Intensive Care and Emergency Medicine, St. Josefs Hospital, Freiburg, Germany

**Keywords:** First responder, Smartphone alerting systems, Out-of-hospital cardiac arrest, Automated external defibrillator, Public access defibrillation, Dispatch centre

## Abstract

**Introduction:**

Out-of-hospital cardiac arrest (OHCA) is a critical medical emergency where rapid access to automated external defibrillators (AED) can significantly improve survival rates. However, there is currently a lack of well-established frameworks and guidelines concerning the optimal placement of AED. Additionally, historical data on the locations of OHCA incidents is often unavailable or incomplete. This study seeks to address these gaps by analyzing the most effective AED placement strategies and evaluating the impact of additional AED locations on suspected OHCA cases. To achieve this, a machine learning (ML) model is developed that relies exclusively on demographic and infrastructural factors, without the need for historical OHCA location data.

**Methods:**

In this data-driven predictive modelling study, 5,076 alerts of suspected OHCA and 95 AED locations in Freiburg were analysed (October 7, 2018, to May 28, 2024). Demographic and infrastructural data were integrated into a three-step approach to identify and prioritize optimal AED placements. A Decision Tree was trained to predict OHCA risk at possible locations, followed by the application of a greedy algorithm to determine AED locations. The models were validated using several performance metrics and historical OHCA data to ensure accuracy. Additionally, different scenarios were evaluated to maximize AED coverage of OHCA incidents.

**Results:**

Optimizing AED placement using predicted data increased coverage from 21.6% to 42.4%, without adding more devices. The ML model’s coverage was only 6.7% lower than that achieved using historical alert data. Adding 19 AEDs (a 20% increase) to the existing network raised coverage to 30.5%.

**Conclusion:**

The findings demonstrate the feasibility of using ML models for AED placement in regions lacking comprehensive historical data. Integrating advanced ML techniques can further refine strategies for AED deployment in urban areas, ultimately improving emergency response effectiveness.

**Trial registration:**

The trial is registered with the German Clinical Trials Register (DRKS, ID: DRKS00016625 15/04/2019 and DRKS00032957 30/10/2023), which is a WHO primary register.

**Supplementary Information:**

The online version contains supplementary material available at 10.1186/s12873-025-01441-3.

## Background

Every year, millions of people worldwide suffer from out-of-hospital cardiac arrest (OHCA) where rapid access to automated external defibrillators (AED) potentially could improve survival rates significantly [1–4]. The American Heart Task Force first formulated the concept of Public Access Defibrillators (PAD) in 1990, calling for the availability of AED in high-traffic areas that can be operated by trained or untrained laypersons in the event of an OHCA [5]. However, AED are rarely used in OHCA [2]. In addition, there are smartphone alerting systems (SAS), which aim to improve the use and availability of AED/PAD [6–8]. SAS are designed to notify nearby registered first responders via an app, enabling them to reach the scene faster than emergency services, initiate life-saving measures, and bring a nearby AED to the patient. Therefore those systems should be linked to AED location registries [9]. A SAS has been implemented in the metropolitan area of Freiburg/Germany (1,531 km^2^, 493,036 inhabitants) in 2018 by the non-profit organisation Region of Lifesavers (“Region der Lebensretter”). Part of the project is to improve AED availability and establish additional AED locations [6, 10]. Generally, the use of AED in easily accessible locations with a high probability of OHCA is recommended [11, 12]. Demographic and infrastructural characteristics can be considered as key factors for the strategic placement of AED in public areas [13–17]. For example, locations with an older population structure, transportation or leisure facilities have been identified in several studies as high-risk areas that could benefit [14–16]. The 2021 European Resuscitation Council (ERC) guidelines recommend two AED per km^2^ [12]. However, generally applicable guidelines or strategies for the placement of AED in public areas are not available yet. Although demographic and infrastructural factors are considered essential in most studies, few models have been developed for the placement of AED [18–20].

The aim of this study is to develop a predictive model based on a machine learning (ML) algorithm for strategic AED placement resulting in highest possible AED coverage in Freiburg, excluding data of preceding locations of OHCA alerts.

## Methods

### Study sample and design

For this data-driven forecast modelling study, the geographic coordinates of 5,076 SAS alerts from the dispatch centre Freiburg (Germany) for suspected OHCA between October 7, 2018 and May 28, 2024 were analysed. Furthermore, the geolocation data (effective May 28, 2024) of 95 AED with 24/7 availability in Freiburg were integrated in the analysis (semi-automatic AEDs).

The demographic data was provided by “The Humanitarian Data Exchange” (Meta, USA, Version 01.07.2019) [21] while the infrastructural characteristics were provided by the Overpass API (DinoTools, Overpy 0.7, 04.12.2023) [22]. A three-step approach was conducted to identify and characterize possible AED locations, predicting the risk for OHCA at the identified locations and prioritize AED placements based on a greedy algorithm.

### Identification and characterization of possible AED locations (Step 1)

**Fig. 1:**
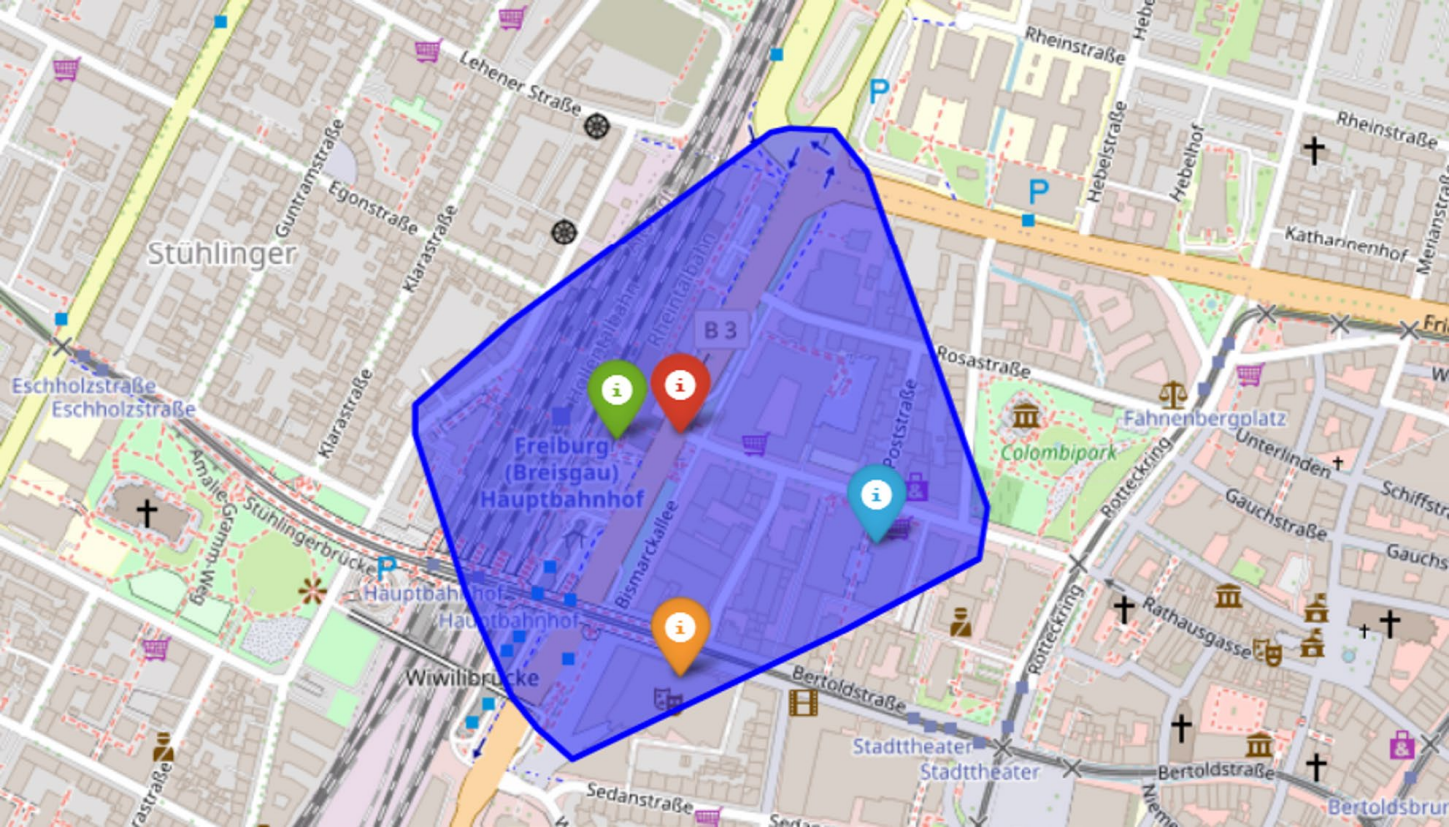
Example of the representation of an isochrone in Freiburg. The red marker symbolizes a road junction, while the blue area represents a three-minute isochrone (walking). Within the isochrone are infrastructural features such as the main train station (green marker), the theatre (orange marker), or parking spaces (blue marker). The isochrone also contains demographic features, such as the number of people living in the isochrone

Based on the study by Berg and colleagues [23], the area of Freiburg was divided into 31,023 isochrones around road intersections, with their centres serving as possible locations for AED (Fig. 1). Each isochrone covers a three-minute walking distance, equivalent to 297 m at a speed of 1.65 m/s [24]. Possible locations are then characterized based on the demographic and infrastructural features of the associated isochrones, which are used as inputs for a Decision Tree Model to predict OHCA alert locations (sex, population density, age structure, public buildings, residential areas/industrial areas, sport facilities, transportation stations and public parking spaces). These characterized features help identify *risk areas* for incidence of OHCA cases (**Supplement**). The target variable for the prediction model is a risk area based on the average number of OHCA alerts per year during the observation period (Table 1).

**Table 1:** Risk areas and the corresponding number of alerts per year

Risk area	Number of alerts per year
1	0
2	(0–1]
3	(1–2]
4	(2–3]
5	(3–4]
6	(4–5]
7	(5–10]
8	(10–20]
9	(> 20)

### Predicting the risk for OHCA at possible AED locations (Step 2)

A Decision Tree was trained to predict the risk area of potential locations for OHCA based on historical data from the dispatch center. By using supervised learning, the model can estimate risk areas using demographic and infrastructural data, even in regions where detailed information is lacking. To improve accuracy, any highly correlated factors were removed during data preparation. All input variables were tested for statistical significance using an F-test, with a threshold value of less than 0.05 [25]. Modelling the raw OHCA alert locations counts directly proved unreliable given the limited and heterogeneous data. We therefore chose to predict a continuous risk score on a scale between 1 and 9 that is based on certain thresholds on the number of alerts per year (Table 1). This proved to be a stable and interpretable approximation of the underlying risk pattern [26, 27].

Two types of models were used to find a balance between simplicity and performance: Simple algorithms such as linear regression and decision trees and the automatic machine learning platform AutoML (H2O Driverless AI), which provides an automated fine-tuning of these algorithms. In the end, the Decision Tree provided the best results with regard to the simplicity-performance trade-off. The data was split into 80% for training and 20% for testing, with five-fold cross-validation to avoid overfitting.

### Greedy prioritization of AED placements (Step 3)

The predictive model classifies 31,023 possible AED locations in Freiburg into risk areas. As the associated isochrones partially overlap, placing an AED at every high-risk OHCA location would result in overlapping coverage and ineffective placements. A greedy-based algorithm is designed to address the goal of finding the appropriate subset of AED locations. A detailed pseudo-code is provided in the **supplement**. The algorithm iteratively places AED at the respective location with the highest risk area. Locations within the 3-minute isochrone of the selected site are removed to avoid redundant coverage. The risk area of locations within the five-minute isochrone is reduced by a constant to balance overlap with newly covered areas, ensuring high-risk locations can still be considered in later iterations. The process repeats until either a fixed number of AED is selected, or a certain risk area is covered. A sensitivity analysis revealed that the choice of the constant for the reduction of the risk level has a marginal effect on the resulting coverage. In the present dataset with a reduction by two results in the largest coverage in the given data set. In scenarios where AED are already installed, potential locations are pre-processed by removing those that fall within a three-minute isochrone of an existing AED or reducing the risk area by the constant of those that fall within a five-minute isochrone of an existing AED.

### Validation and scenarios

The scenarios used in the validation are presented in Table 2. The study compares the coverage of historical alerts when AED are installed based on the historical alerts versus AED placement using the predictive model. In this comparison, Scenario 1 represents installation using the predictive model, while Scenario 2 uses historical OHCA dispatches. In both scenarios, 95 new AED are installed, the same number as currently installed, to ensure a fair comparison to the current status (Scenario 0). The primary metric for validation is the percentage of historical alerts covered, defined by the presence of an AED within 300 m of the event. This analysis allows for a comprehensive comparison of different AED placement strategies and their effectiveness in improving emergency response coverage. Scenarios A, B, and C, along with the current AED status (Scenario 0), evaluate the impact of additional AED placement using a predictive model. Scenario A assumes a 20% increase in the number of AED, while in Scenario B and C AED are placed to cover a certain risk area (risk area 5 or risk area 3, see Table 1). These scenarios evaluate how an increase in the number of AED increases the coverage of historical OHCA alerts.

**Table 2:** Scenarios used in the investigation

Scenario	Considering installed AED	Termination criteria	Data basis
Scenario 0	Yes	-	-
Scenario 1	No	*N* = 95 AED	Predictive model
Scenario 2	No	*N* = 95 AED	Historical alerts
Scenario A	Yes	*N* = 19 AED	Predictive model
Scenario B	Yes	Risk area 5	Predictive model
Scenario C	Yes	Risk area 3	Predictive model

### Statistical analysis and Ethics

The following software tools were used in this study: PyCharm 2024.1.1 (JetBrains) served as the integrated development environment, while Python (Versions 3.12.1 and 3.11.6, scikit-learn Version 1.5.2, Python Software Foundation) was utilized for programming. Docker Desktop 4.30.0 (Docker Inc.) was used to operate a Docker container, and OpenRouteService (ORS) vOPENAPI_3_0, executed within a Docker container via ors-docker compose (HeiGIT gGmbH, Heidelberg, Germany), was used to generate isochrones and calculate distances. Additionally, Visual Studio Code (Version 1.89, Microsoft) was also utilized. Statistical analyses were conducted using ANOVA, Variance Inflation Factor (VIF), Mean Squared Error (MSE), and Mean Absolute Error (MAE). A significance level of *P* < .05 was considered statistically significant.

The study has been approved by the Ethics Committee of the Albert-Ludwigs-University Freiburg (482/18 and 23-1450-S1). The research data was used based on the End User License Agreement (EULA), which was agreed by the participating first responders upon registration. Informed consent to participate was obtained from all of the participants. The data protection concept has been reviewed and approved by the state data protection officer. The trial is registered with the German Clinical Trials Register (DRKS, ID: DRKS00016625 15/04/2019 and DRKS00032957 30/10/2023), which is a WHO primary register.

## Results

### Comparison of coverage based on the predictive model and OHCA data (Scenario 1, 2)

**Fig. 2:**
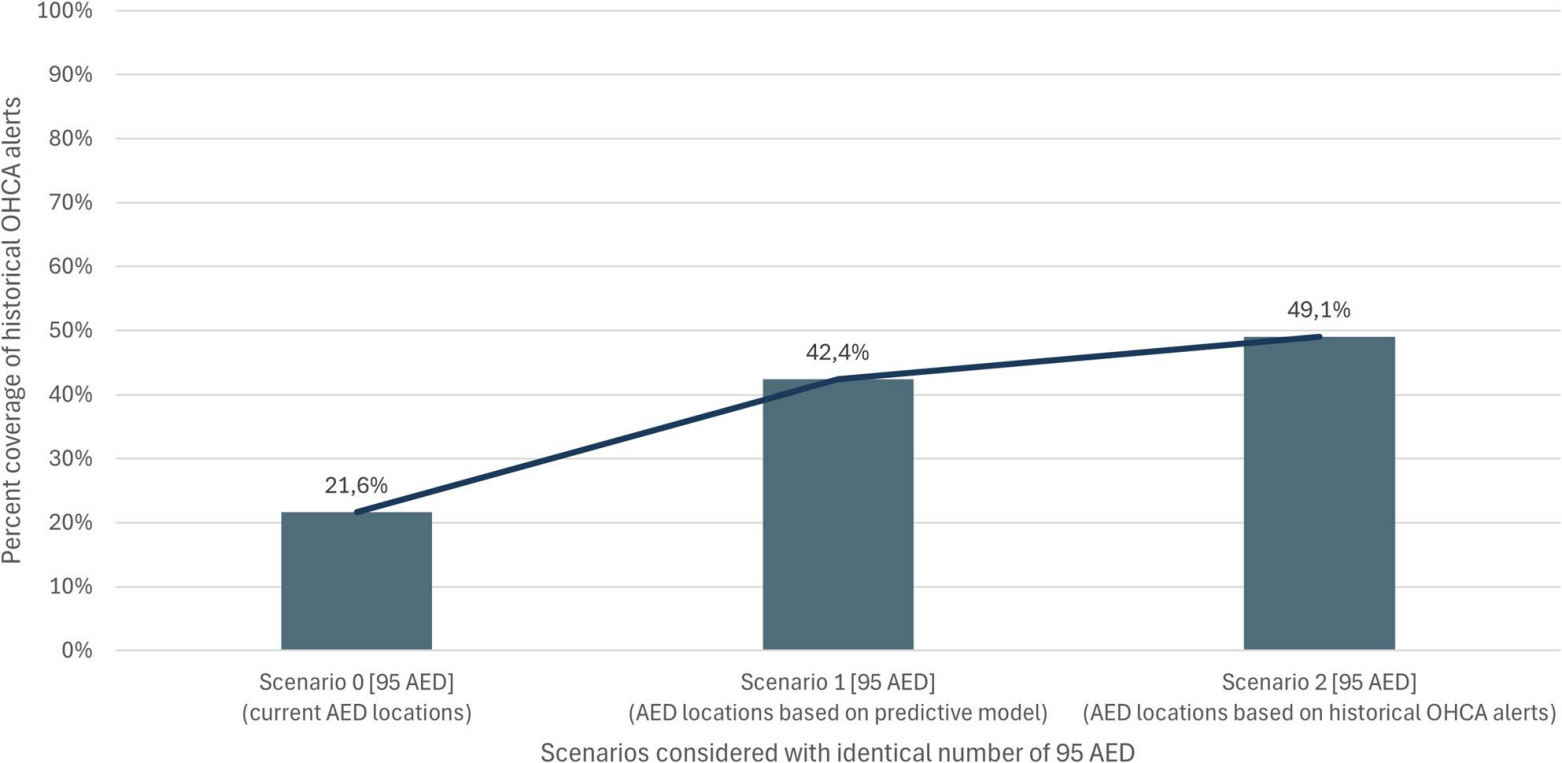
Coverage of historical OHCA alerts in Freiburg with current AED locations (Scenario 0), with AED locations based on predictive ML model (Scenario 1) and with AED locations based on historical alert data (Scenario 2)

In the current situation, 95 AED are installed in Freiburg, providing 21.6% coverage (Scenario 0). In Scenario 1 and 2, the same number of AED were installed, but in different locations. Scenario 1 used the predictive model, while Scenario 2 relied on historical alert data. Coverage reached 42.4% in Scenario 1 and 49.1% in Scenario 2, showing a nominal difference of 6.7% (Fig. 2). Both methods improve AED coverage, with the predictive model yielding slightly less favourable results than the historical data.

**Fig. 3:**
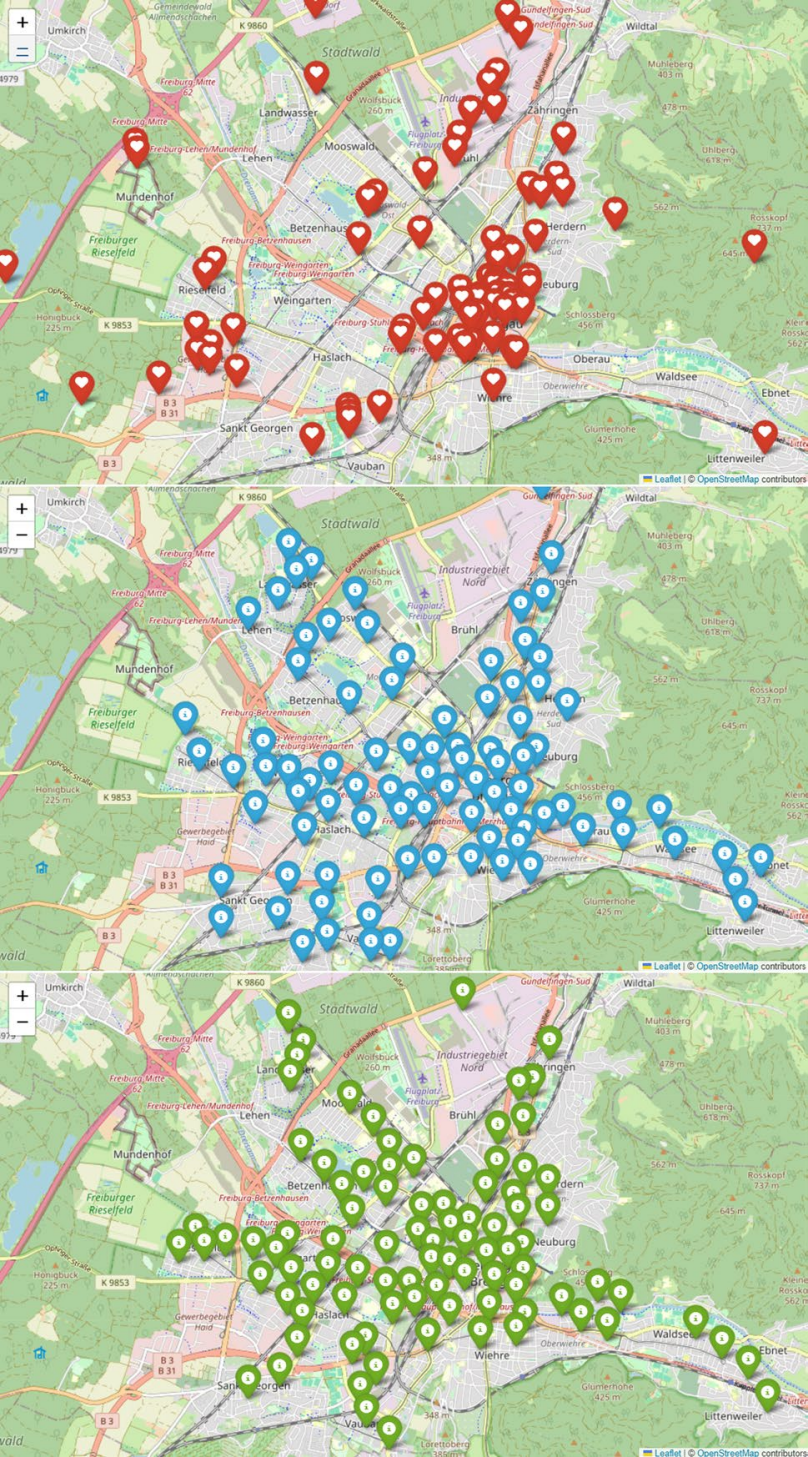
Placement of AED in Freiburg. Scenario 0 (= current AED locations) (red), Scenario 1 (blue) with AED locations based on predictive ML model, Scenario 2 (green) with AED locations based on historical alert data

Figure 3 focuses on a section of downtown Freiburg, where the majority of the 95 AED are installed. In some cases, AED are placed outside the depicted area. The current distribution shows a concentration in the city centre and a few residential areas. In contrast, both Scenario 1 and Scenario 2 demonstrate a more balanced AED placement, covering the city centre as well as multiple residential areas. This leads to a more even distribution of AED compared to the existing situation.

### Expanded AED coverage considering current AED locations (Scenario A, B, C)

**Fig. 4:**
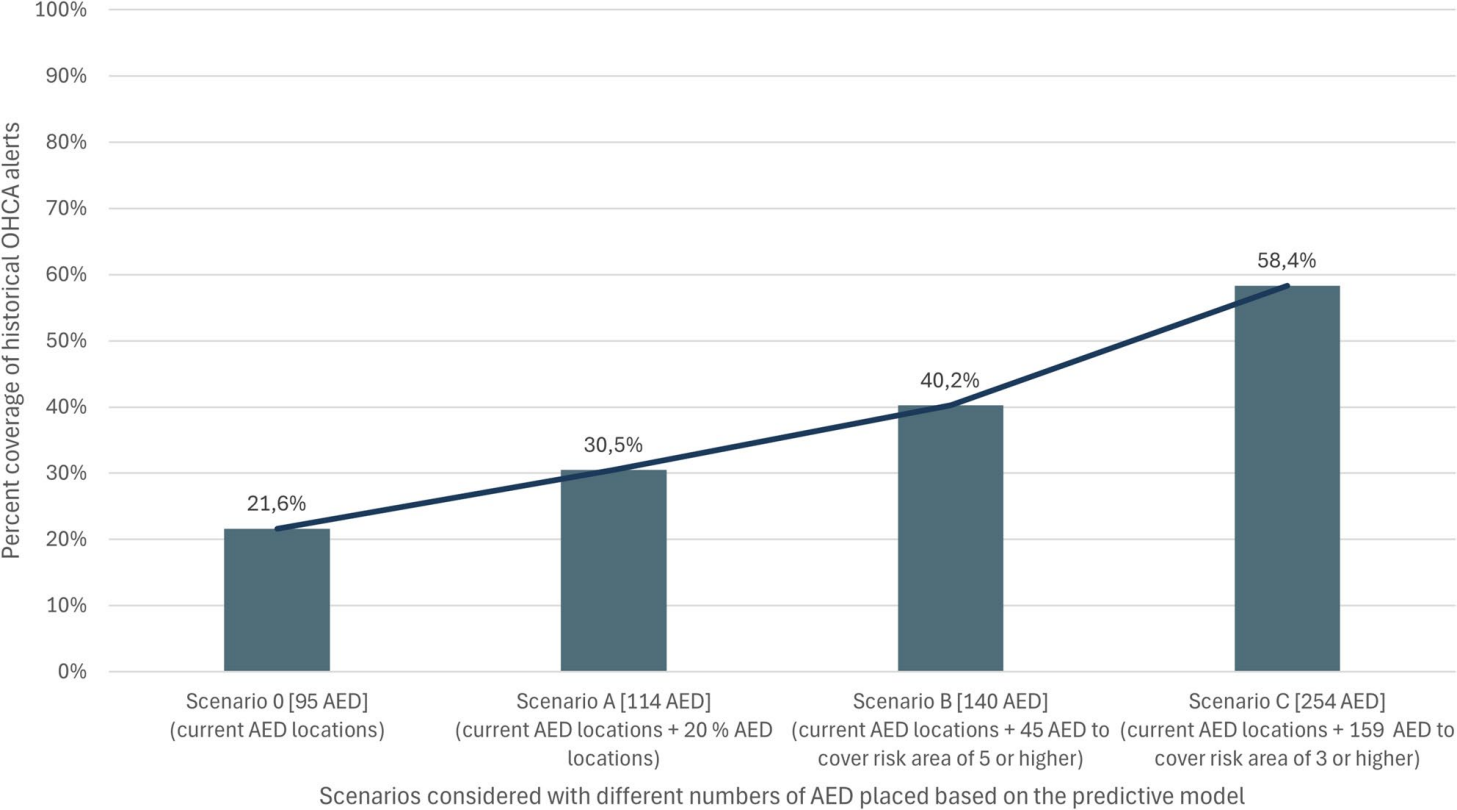
Coverage of historical alerts in Freiburg considering current AED locations (95 AED) already installed. Scenario 0 Shows coverage with current AED locations. Scenario A shows coverage with additional 20% (= 114) AED locations placed by predictive model. Scenario B and C show coverage of historical alerts and needed number of AED when risk area 5 (Scenario B) or risk area 3 (Scenario C) is covered by AED locations

In Scenario A with 20% higher number of AED (total number of 114 AED) placed by the predictive model, the coverage raises to 30.5% (Fig. 4).

In Scenarios B and C, the required number of AED was analyzed based on the coverage of different risk areas with AED locations. For Scenario B, which targets risk area 5 (characterized by 2–3 alerts per year), it was determined that 140 AED would be necessary, resulting in a coverage rate of 40.2% for historical alerts. In Scenario C, focusing on risk area 3 (characterized by 1–2 alerts per year), the analysis indicated a requirement of 254 AED, achieving a coverage rate of 58.4% for historical alerts.

Of the 5,076 alerts during the observation period, only 1,098 could be covered with the current AED locations (three minutes walking distance). This rises to 2,962 alerts in Scenario C. All scenarios used in the validation are presented in Table 2.

## Discussion

The findings of this study align with existing literature, which suggests that AED placements based solely on local initiatives without strategic planning are less effective [14, 28]. The study’s scenario analysis demonstrates that increasing the number of strategically placed AED can significantly enhance coverage. A 20% increase in AED installation results in a 41% improvement in coverage, underscoring the efficacy of well-planned AED placements (Scenario A). The additional benefits or utility gained from more AED locations decrease in Scenarios B and C compared to the initial benefits observed in Scenario A. As a result, the marginal benefit of each additional AED diminishes, even though the overall coverage improves. These findings suggest that while expanding AED coverage is beneficial, cost-effectiveness also has to be taken into account to avoid over-saturation in low-risk areas. There are multiple strategies for AED placement released by the European Resuscitation Council (ERC) and the American Heart Association (AHA), however they also address a relevant knowledge gap in this field implying continued research [12, 29, 30]. Although the accessibility of AED was not the primary focus of this study, it is important to highlight that AED should be available 24/7, clearly marked, and easily accessible to ensure prompt use in emergencies [31, 32]. The approach of this study is designed to be generally applicable, assuming that temporarily accessible AEDs cannot be relied upon, particularly during nighttime or off-hours. Consequently, only AEDs with 24/7 availability were considered to ensure consistent and realistic coverage assessment.

It is conceivable, that there is no way for a 100% AED coverage for OHCA scenarios. However, to enable rapid defibrillation in a very high proportion of OHCA cases it may be helpful to combine strategic placement of AED with a current concept to integrate AED drones [33]. While most common approaches focus on data analysis of historical OHCA aim to identify and equip high-risk areas [13], this strategy may be influenced by various factors, such as the consideration of historical alert data of OHCA [34], demographic and infrastructural factors [35], geographical and temporal availability of AED [15] or the daytime population [13, 14]. Current studies are increasingly focusing on the limited or unlimited availability of AED at different times of the day [28]. The spatial restrictions indicate that AED cannot be available around the clock. This factor should be considered when strategically planning the availability of AED.

The analysis highlights population density as the most important factor influencing OHCA location likelihood, confirming previous research on the correlation between high traffic areas and OHCA risk. Although age-related data were unavailable for this study, there is a suggested trend between regions frequented by older individuals and elevated OHCA risk [36, 37]. Additionally, infrastructural features like public buildings and transportation hubs, also play an essential role, as these areas attract large transient populations, further increasing OHCA alert risk.

The comparison between AED placement based on historical data and the predictive model reveals only a minor difference in coverage (6.7%), affirming the model’s reliability. This demonstrates that predictive ML models based on demographic and infrastructural data may be effectively applied in regions lacking historical OHCA location data, offering a robust alternative for strategic AED planning.

The objective in everyday practice is to ensure that every OHCA alert (Operation via Smartphone Alerting System) is equipped with an AED. Consequently, the primary goal is not solely to address cases that later confirm to be OHCA, but to have an AED available for every SAS operation, thereby covering all suspected cases of OHCA.

## Conclusion

This study highlights the role of integrating demographic and infrastructural characteristics into ML models for the optimal placement of AED. The findings may indicate that these models provide coverage nearly equivalent to that achieved using historical OHCA location data, affirming their reliability and effectiveness. Additionally, the strategic addition of AED greatly enhances coverage, particularly in high-risk areas identified through the ML model.

The study offers a robust methodological framework for AED placement in urban settings, emphasizing data-driven decision-making in public health initiatives. Future applications may be further strengthened by incorporating advanced ML techniques to identify complex patterns within extensive datasets, enabling more accurate predictions of ideal AED locations.

### Limitation

Time-related factors, such as AED availability at different times of day, operational hours, and time-dependent characteristics like population density, were not included in the current model. Additionally, distance calculations were limited to horizontal distances, excluding vertical dimensions within buildings and calculation was based on walking speed. Vertical accessibility is likely of minor relevance in this analysis, as only AEDs with 24/7 availability were considered, which are typically installed at ground level rather than on upper floors. It can be assumed that the first responders run and can therefore cover the distance in less time. Whereas traffic should also be taken into account.

The greedy algorithm used in this study tends to find local rather than global optima, which may result in suboptimal outcomes, especially in complex problems like AED placement. Greedy algorithms are known to be quick and simple to implement but can miss better global solutions [38]. Introducing an improvement heuristic could enhance optimization.

The predictive model relies on a limited amount of labelled training data, restricting its ability to generalize. This effect is amplified when predicting OHCA alert locations per isochrone, as the dataset contains a high proportion of zero events. Insufficient data may cause the model to overfit specific patterns, struggling to adapt to new, unseen scenarios. Expanding the training data would improve generalizability and enable a more detailed validation explicitly accounting for spatial or temporal aspects. In addition, while several predictors already capture key spatial characteristics, we did not formally test for residual spatial autocorrelation. Given the model’s explanatory performance, however, any remaining spatial dependence is expected to be limited.

Lastly, model validation is necessary when applied to other regions. Testing predictions in different areas with known data would assess the model’s robustness and ensure its applicability across varying geographical and demographic contexts. The presented approach represents an initial framework for strategic AED planning and invites further research and methodological advancement to evaluate and adapt the concept in different urban and regional settings.

## Supplementary Information

Below is the link to the electronic supplementary material.

Supplementary Material 1

## Data Availability

Reasonable requests to provide raw data material will be assessed by the board of Region of Lifesavers (Region der Lebensretter) organization.

## Declarations

### Language check

During the preparation of this work the authors used Microsoft Word 365 Editor and OpenAI ChatGPT 2024 to check language and readability. After using this service, the authors reviewed and edited the content as needed and take full responsibility for the content of the publication.

## Abbreviations

AED: Automated External Defibrillator
ML: Machine Learning
PAD: Public Access Defibrillation
SAS: Smartphone Alerting System
OHCA: Out-of-hospital cardiac arrest
WHO: Word Health Organization
