# Supplementary material for: Identifying optimal locations for automated external defibrillators (AED) in Freiburg: development and validation of a machine learning model based on demographic and infrastructural data
Source: BMC Emerg Med. 2025 Dec 13;26:19. doi: 10.1186/s12873-025-01441-3 (PMC12817458; doi:10.1186/s12873-025-01441-3)

**Supplement**

1. Demographic and infrastructural characteristics evaluated in literature

|  | Demographic char | | | | Infrastructural char | | | | | | |
| --- | --- | --- | --- | --- | --- | --- | --- | --- | --- | --- | --- |
|  | Population density | Age structure | Daytime population | Gender | Public buildings | Residential areas vs. Industrial areas | Educational institutions | Sports facilities | Transport hubs | Parking | 24/7 AED vs. time restricted availability |
| [14] |  |  | x | x | x | x | x | x | x |  | x |
| [18] | x |  |  |  | x | x | x | x | x | x |  |
| [36] | x |  |  | x | x | x | x |  | x | x |  |
| [15] | x | x |  | x | x | x |  |  | x |  | x |
| [13] | x | x |  | x | x |  | x | x | x |  |  |
| [31] | x | x |  |  | x | x |  | x |  |  | x |
| [4] |  |  |  |  | x | x | x | x | x |  | x |
| [32] | x |  |  |  | x |  | x | x | x |  |  |
| [19] | x |  | x |  |  | x |  |  |  |  | x |

1. Results of ANOVA with F-value and p-value for each feature:

| **Feature** | **F-value** | **p-value** |
| --- | --- | --- |
| Population | 18371.919247 | 0.000000e+00 |
| Residential areas | 8089.140439 | 0.000000e+00 |
| Parking | 7284.348433 | 0.000000e+00 |
| Parks | 3963.455311 | 0.000000e+00 |
| Theatres | 3586.365953 | 0.000000e+00 |
| Religious sites | 3257.738356 | 0.000000e+00 |
| Markets | 2934.168049 | 0.000000e+00 |
| Sports facilities | 2857.015011 | 0.000000e+00 |
| Industrial areas | 2775.149932 | 0.000000e+00 |
| Nursing homes | 1629.696048 | 0.000000e+00 |
| Community centres | 1359.730334 | 7.762440e-290 |
| Art facilities | 1351.066354 | 4.734100e-288 |
| Cinemas | 1078.634422 | 1.316034e-231 |
| Train stations | 903.385446 | 5.621491e-195 |
| Casinos | 834.398665 | 1.737839e-180 |
| Social institutions | 151.253217 | 1.164251e-34 |
| Exhibition centres | 30.405104 | 3.540962e-08 |
| Sports arenas | 8.838138 | 2.952832e-03 |

1. Results of the variance inflation factor (VIF) of each feature

| **Feature** | **VIF** |
| --- | --- |
| Population | 6.903212 |
| Train stations | 1.264634 |
| Parking | 5.888289 |
| Art facilities | 1.195090 |
| Casinos | 1.257098 |
| Cinemas | 1.193095 |
| Community centres | 1.295259 |
| Theatres | 1.482207 |
| Exhibition centres | 1.030048 |
| Markets | 1.321403 |
| Religious sites | 1.495396 |
| Nursing homes | 1.291831 |
| Social institutions | 1.084918 |
| Sports facilities | 2.375234 |
| Sports arenas | 1.123405 |
| Residential areas | 7.342220 |
| Industrial areas | 2.166168 |
| Parks | 2.934620 |

1. Importance of input features / characteristics in the decision tree model

| **Feature** | **Importance** |
| --- | --- |
| Population | 0.6240 |
| Theatres | 0.0964 |
| Nursing homes | 0.0320 |
| Train stations | 0.0318 |
| Parks | 0.0307 |
| Parking | 0.0286 |
| Industrial areas | 0.0280 |
| Markets | 0.0234 |
| Religious sites | 0.0213 |
| Community centres | 0.0205 |
| Sports facilities | 0.0169 |
| Art facilities | 0.0139 |
| Casinos | 0.0093 |
| Cinemas | 0.0071 |
| Residential areas | 0.0067 |
| Social institutions | 0.0046 |
| Sports arenas | 0.0042 |
| Exhibition centres | 0,0006 |

1. Validation

The results for linear regression, decision trees, and H2O were:

Mean Squared Error (MSE): 1.3433, 0.9921, and 0.7298

Mean Absolute Error (MAE): 0.8400, 0.6474, and 0.5672

1. Greedy selection of AED Locations


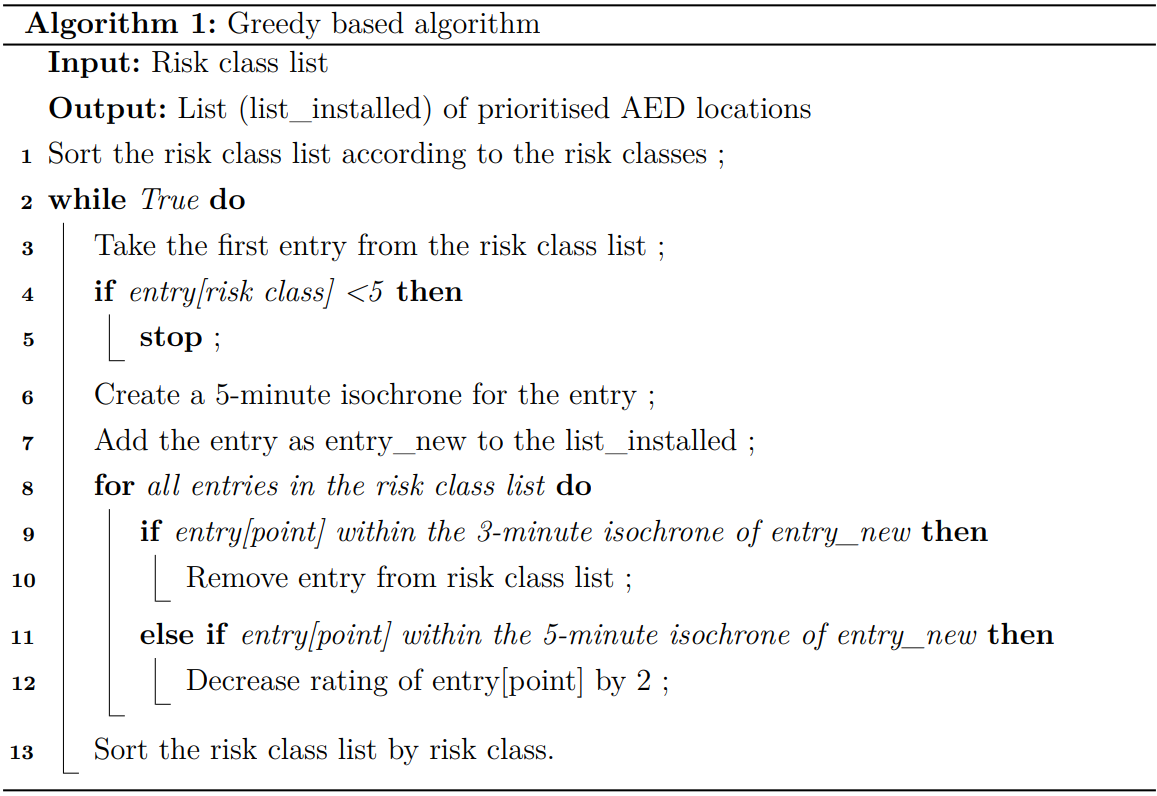


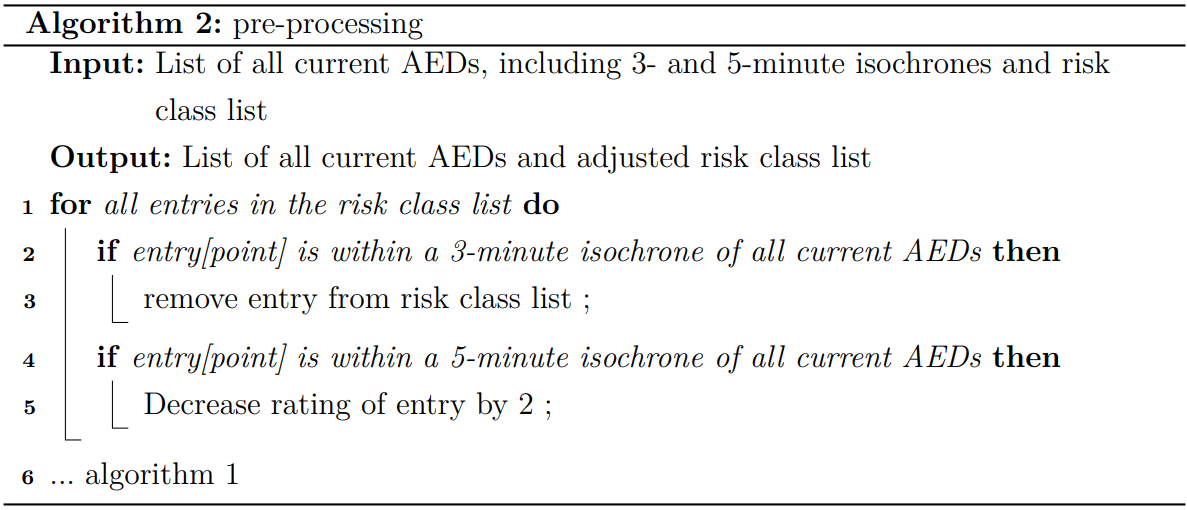

Supplement: Supplementary file 1 — Supplementary Material 1 [file 12873_2025_1441_MOESM1_ESM.docx]
